# Phase I/II study of induction chemotherapy plus concurrent chemotherapy and SMART-IMRT-based radiotherapy in locoregionally-advanced nasopharyngeal cancer

**DOI:** 10.3892/ol.2013.1137

**Published:** 2013-01-15

**Authors:** TING-YONG FAN, JUN XING, JIE LU, TONG-HAI LIU, MIN XU, YING-JIE ZHANG, QIAN SHAO, JIAN-BIN LI, JIN-MING YU

**Affiliations:** 1 School of Medicine, Shandong University, Jinan, Shandong 250012;; 2 Shandong Cancer Hospital and Institute, Jinan, Shandong 240117, P.R. China

**Keywords:** nasopharyngeal cancer, induction chemotherapy, concurrent chemoradiotherapy, SMART-IMRT, cisplatin

## Abstract

This study aimed to evaluate the efficacy, toxicity and tolerability of simultaneous modulated accelerated radiation therapy (SMART)-intensity modulated radiotherapy (IMRT) plus cisplatin and 5-fluorouracil (5-FU) chemotherapy for patients with advanced nasopharyngeal cancer (NPC). Forty-five patients with stage II–IV NPC, determined by the American Joint Committee on Cancer system, were treated with prescribed doses of 72 Gy total to the gross tumor volume, 60 Gy to the clinical target volume and metastatic nodal station, and 54 Gy to the clinically-negative neck region. Before radiotherapy, two cycles of cisplatin (30 mg/m^2^/day on days 1–3) plus 5-FU (400 mg/m^2^/day on days 1–5) were delivered every three weeks for two cycles. Patients received two cycles of cisplatin (30 mg/m^2^ day on days 1–3) every three weeks during radiotherapy. In addition, two cycles of cisplatin and 5-FU were given after radiation. All patients completed the prescribed radiotherapy and all scheduled cycles of chemotherapy. Thirty of the 45 patients (66.6%) had a complete response at the end of treatment. Grade 3 mucositis occurred in 4/45 patients (8.8%) and grade 3 dermatitis occurred in 5/45 (11.1%) during radiotherapy. Grade 3 neutropenia occurred in 6/45 (13.3%) during concurrent chemotherapy. There was no treatment-related mortality. After a median follow-up time of 51 months, only three patients’ treatments had failed. Local and distant failure rates were 1.5 and 3.0%, respectively. SMART-IMRT plus cisplatin and 5-FU chemotherapy showed promising activity with manageable toxicity. It is a feasible regimen and improves locoregional disease control.

## Introduction

Nasopharyngeal carcinoma (NPC) is a non-lymphomatous squamous cell carcinoma that originates from the epithelial lining of the nasopharynx (1,2). Pathologically, NPC presents in varying degrees of differentiation and is frequently located on the pharyngeal recess posteromedial to the medial crura of the eustachian tube (3). NPC is not common in most countries; its age-adjusted incidence for both genders is <1 per 100,000 (4). According to the International Agency for Research on Cancer, there were ∼84,400 cases of NPC and 51,600 related mortalities in 2009 (4).

The histological types of NPC are defined as either squamous cell carcinoma or non-keratinizing carcinoma. They are further classified into either differentiated or undifferentiated carcinoma (5). The three well-established etiological factors for NPC include genetically-determined susceptibility, early-age exposure to chemical carcinogens (e.g., salted fish in southern China), and Epstein-Barr virus (EBV) infection (6).

Irradiation is the primary therapeutic modality for NPC due to its anatomical location and high radiosensitivity. Treatment of early-stage disease with radiotherapy alone usually results in successful control (7) but the response of locoregionally-advanced NPC with radiotherapy alone is unsatisfactory, with a 5-year overall survival (OS) rate of 87–96% for stages I–II and 67–77% for stages III–IV (1). This is significant because according to the 6th American Joint Commission on Cancer (AJCC) staging system, 60–70% of patients present with stage III–IV disease at the time of diagnosis (8).

Concurrent chemoradiotherapy with adjuvant chemotherapy has been deemed the standard of care for advanced NPC since 1998 (9). This was initally due to the Intergroup 0099 study, which found a 31% increase in 3-year OS. Recently, seven randomized-control phase III clinical trials (comparing chemo-radiation with radiotherapy alone) confirmed the effects of additional chemotherapy on the survival of patients with advanced NPC. Three of these trials compared concurrent chemoradiotherapy with radiotherapy alone (10–13), while the other four trials adopted the concurrent chemoradiotherapy plus adjuvant chemotherapy regimen (8,9,14,15), analogous to that of the Intergroup 0099 study. The latter four trials were, however, unable to rule out the contribution of adjuvant chemotherapy since radiotherapy alone was regarded as the control. Furthermore, three phase III trials, in which adjuvant chemotherapy was applied alone (16) did not show a positive effect on the OS of patients with advanced NPC. It is, therefore, unclear whether adjuvant chemotherapy contributes to an additional survival benefit over concurrent chemoradiotherapy in advanced NPC.

Currently, radiotherapy is the standard treatment for NPC. Unfortunately, it leads to the development of undesirable complications, primarily due to the anatomical location of the tumors at the base of the skull where they are in close proximity to radiation dose-limiting organs, such as the brain stem and spinal cord. With the advent of intensity-modulated radiotherapy (IMRT), radiation beams can now be modulated so that a high dose can be efficiently delivered to the tumor while limiting the dose to the surrounding normal tissue (17). Except for the conformal distribution of radiation, IMRT further exploits accelerated forms of radiotherapy, such as simultaneous modulated accelerated radiation therapy (SMART), in which different doses are simultaneously delivered to different target lesions in an overall shorter treatment time (18,19).

With the advantage of IMRT, the control of locoregional NPC has been substantially improved and the development of distant metastases is now the main cause of treatment failure (20). Further improvements in systemic control of advanced NPC using concurrent chemotherapy are likely, despite the drug-related toxic effects. Meanwhile, it is pivotal to address the issue of neo-adjuvant and adjuvant chemotherapy. In the present study, a retrospective trial was undertaken to determine the efficacy of concurrent chemoradiotherapy plus adjuvant and neo-adjuvant chemotherapy to appraise the contribution of chemotherapy and SMART-IMRT-based radiotherapy in locoregionally-advanced NPC.

## Materials and methods

### Participants and pre-treatment evaluation

From January 2005 to December 2007, 45 NPC patients between stages IIA and IVa (AJCC, 7th edition, 2005) were treated with SMART-IMRT, combined with concurrent chemotherapy and adjuvant chemotherapy following an initial induction chemotherapy regimen. Eligibility criteria for this study were: histologically-confirmed, locoregionally-advanced stage IIA to IVB NPC [World Health Organization (WHO) histopathological type I–III (2)], no previous history of chemotherapy or radiotherapy, no evidence of distant metastases, age 17 to 75 years, an Eastern Cooperative Oncology Group (ECOG) performance status of 0 to 1 or Karnofsky performance score (KPS) ≥70, white blood cells ≥4,000/ml, platelets ≥100,000/ml, serum creatinine ≤1.6 mg/dl or 24-h calculated creatinine clearance ≤60 ml/min, ALT and/or AST ≤40 U/l and no limitation of co-morbidities for the therapeutic protocol. The patients with locoregionally-advanced NPC were excluded if they refuted the entire therapeutic protocol or reported a previous history of other human cancers.

The pre-treatment evaluation consisted of a complete medical history and physical examination, fiber optic endoscopic examination, routine blood counts and serum biochemistry. In all patients, a head and neck CT and/or magnetic resonance imaging (MRI) were used to accurately evaluate the extent of the primary tumor and regional lymph nodes. In addition, chest radiography and abdominal ultra-sonograph were routinely performed. In the clinical trial, positron emission tomography (PET)/CT was suboptimal for the assessment and bone scintigraphy was involved as indicated. All patients in this study explored the potential risks and benefits of SMART-IMRT concurrent with cisplatin-based chemotherapy. The protocol was approved by the institutional ethics committees of the individual participating centers (Shandong Province Oncology Hospital, Jinan, Shandong, China). The trial was monitored by an independent Data Monitoring Committee composed of radiation oncologists, medical oncologists and statistical consultants. All patients provided written informed consent.

### Radiotherapy treatment

Patients were immobilized from the head to shoulders in the supine position using a thermoplastic mask. Pre-treatment planning CT with serial 3 mm slices from the head down through the top of the aortic arch was obtained and the images were transmitted to Pinnacle 8.0 for further analysis. Target volumes were delineated on the treatment planning CT images; lesion location and body position by CT scan was re-evaluated at four weeks after radiation. In certain cases, the planned volume was modified, due to variations in target volume and body contour.

The gross tumor volume (GTV) was defined by a combination of imaging studies (such as CT, MRI or PET), clinical information, endoscopic findings and the initial physical examination. GTV included the nasopharyngeal primary, retropharyngeal lymphadenopathy and all gross nodal disease. Gross nodal disease was defined as any lymph nodes that were histopathologically confirmed as metastasis at the routine drainage regions.

The clinical target volume (CTV) was characterized as all potentially gross and microscopic involvements of NPC, which was divided into high-risk CTV (CTV1) and low-risk CTV (CTV2). CTV1 was contoured as the GTV plus margin for potential microscopic spread, including the entire nasopharynx, skull base, clivus, inferior sphenoid, posterior ethmoid, posterior maxillary antrum and nasal cavity, pterygopalatine fossa, retropharyngeal nodal space and parapharyngeal space. The CTV1 for primary and nodal disease was a concentric volume entirely encompassing the GTV with an additional 5.0–10.0 mm margin. CTV1 for lymphonode disease included the first echelon nodal areas. When level II lymphonodes were grossly involved, ipsilateral levels I and III were considered as CTV1. Furthermore, CTV2 was characterized as the volume involving low-risk subclinical disease. In some cases, grossly negative lymphonode, retropharyngeal, and level II lymph node areas were regarded as CTV1 and the remaining nodal regions (III, IV and V) were regarded as CTV2.

Tumor volumes were contoured with an extra margin of at least 5.0 mm to accommodate variations with the patients’ set-up, and were defined as the planning target volume (PTV). The PTV was practical for the clinical experience of radiologists, but presented a dilemma: an inadequate irradiation treatment dose vs. impairments of fatal organs or tissues. The various nodal levels in the neck were delineated according to the recommendation by an experienced radiologist. In addition, the critical normal tissues, including the brain stem, spinal cord, eye globes, optic nerves, chiasm, parotid glands and lens, were contoured as ‘organs at risk’ (OARs). Whenever possible, MRI images were fused with CT images to delineate the target volumes and the surrounding critical normal structures.

In our institution, IMRT guidelines using SMART techniques were developed for the treatment of head and neck cancers. According to the guidelines, daily fractions of 2.2 and 2.0 Gy were prescribed to the PTV1 and PTV2 with a total dose of 72 and 60 Gy, respectively, in 30 fractions over six weeks. The planning goal was as follows: the prescription dose was to encompass at least 95% of the PTV, no more than 20% of the PTV was to receive more than 110% of the prescribed dose, no more than 1% of the target volume was to receive less than 93% of the prescribed dose, and no more than 1% of the tissue outside the PTV was to receive more than 110% of the prescribed dose. For the OARs, the maximum-tolerated dose was as follows: 54 Gy to the brain stem, optic nerve and chiasm; 45 Gy to spinal cord; and 8 Gy to the lens. As a parallel structure, up to 50% of the parotid glands could receive no more than 30–35 Gy. The total parotid volume was the sum of right and left parotid volumes. SMART was delivered using 6 MV photons generated by a linear accelerator with Millennium 120 MLC (Varian Medical Systems, Palo Alto, CA, USA).

**Figure 1:**
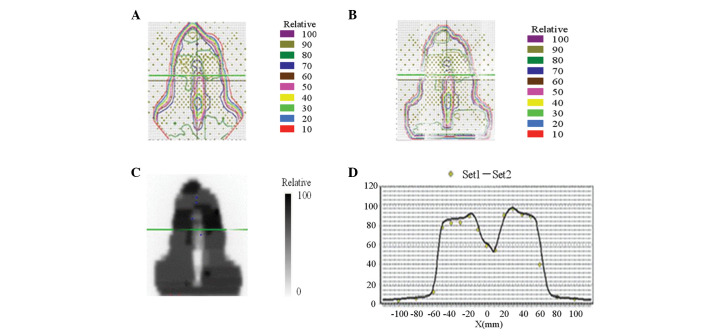
Examples of dose distribution curves of target volume. (A) Practical dose distribution curve. (B) Planned dose distribution curve. (C) A comparison between practical and planned dose distribution curves. (D) Contrast curve of measured vs. calculated irradiation dose. Yellow dots represent measured values and the black line represents calculated values.

As a pre-treatment dose-verification method, the Pin-Point Ionization Chamber (for absolute point dose) and 2D film dosimetry (for dose distribution) were performed. The set-up verification was performed using portal vision and confirmed by the physician every day before treatment. Examples of dose distribution curves of target volume are illustrated in Fig. 1.

### Chemotherapy treatment

All patients recruited into this study were assigned to receive the entire therapeutic protocol consisting of six cycles of a step-by-step radio-chemotherapy. A total cycle of chemotherapy consisted of two cycles of inductive, two cycles of concurrent and two cycles of adjuvant chemotherapy. Inductive and adjuvant chemotherapeutic regimens consisted of cisplatin (30 mg/m^2^/d, i.v. infusion, days 1–3) and 5-FU (400 mg/m^2^/d, i.v. infusion, days 1–5) every three weeks for two cycles. Concurrent chemotherapy was performed alongside SMART-IMRT and consisted of cisplatin alone (30 mg/m^2^/d, i.v. infusion, days 1–3), given every three weeks for two cycles. In this study, a patient’s concurrent chemotherapeutic regimen was chosen by the medical oncologists. Side effects that were ascribed to chemotherapy did not limit the compliance of treatment and all patients fulfilled the therapeutic regimen as planned.

Dose modifications during chemotherapy were based on the nadir blood counts and interim toxic effects. Cisplatin was decreased to 20 mg/m^2^ if the neutro-phil count was 1,000–1,500 cells i.v., the platelet count was 50,000–75,000/*μ*l or the creatinine clearance was 40–60 ml/min. Cisplatin was decreased to 10 mg/m^2^ if the neutro phil count was <1,000 cells/*μ*l or the platelet count was <50,000/*μ*l. Chemotherapy was stopped completely if the creatinine clearance was <40 ml/min or if grade 3 or higher neuro toxicity or ototoxicity developed. 5-FU was decreased to 300 mg/m^2^ if grade 2 (or 200 mg/m^2^ if grade 3) mucositis or diarrhea occurred. Chemotherapy was paused indefinitely if any grade 4 toxic effects developed.

### Toxicity evaluation and follow-up

Throughout the therapeutic process, toxic effects were assessed weekly, both during and after the completion of adjuvant chemotherapy and at subsequent predefined intervals. One month after radiation, all patients underwent a cranial CT scan. After completion of treatment, patients were checked every three months for two years, and every six months thereafter. The patients’ therapeutic responses to radiotherapy were categorized in accordance with the Response Evaluation Criteria in Solid Tumors (RECIST). Chemotherapy-related toxic effects were graded in accordance with WHO criteria. Radiotherapy-related toxic effects were graded in accordance with the Radiation Therapy Oncology Group (RTOG) criteria.

All local recurrences were diagnosed with fiber optic endoscopy and biopsy and/or MRI scan of the nasopharynx and skull base to determine the degree of bone erosion and soft tissue swelling. Regional recurrences were diagnosed by clinical examination of the neck and, in the rare cases, by fine needle aspiration or an MRI scan of the neck. Distant metastasis was diagnosed by clinical symptoms, physical examinations and imaging methods that included chest radiography, bone scan, MRI or CT. PET was also recommended when locoregional recurrence was suspected on CT or MRI. Whenever possible, salvage treatments (including re-irradiation, chemotherapy, and surgery) were given to patients after documented relapse or after persistent disease, in accordance with the standard practice of each center.

### Statistical analysis

A complete response (CR) was defined as the complete regression of all gross or microscopic tumors. A partial response (PR) was defined as >50% regression of all measurable tumors. Treatment failure was recorded as a local failure, regional failure or distant metastasis. OS was defined from the date of randomization initiation to death from any causes. Progression-free survival (PFS) was defined from the date of randomization initiation to treatment failure or death from any cause, whichever occurred first. The toxicity variation in the patients treated with radio-chemotherapy were assessed using the Chi-square or Fisher’s exact test. An independent t-test was used to evaluate the correlation between dose-volumetric parameters and the toxicity.

## Results

### Immediate and long-term treatment outcomes

Forty-five patients with locoregionally-advanced NPC were selected for this study. All cases were histopathologically confirmed as nasopharyngeal squamous cell carcinoma (NPSCC), including 43 poorly-differentiated, 1 moderately-differentiated and 1 well-differentiated NPSCC. All patients were categorized according to the AJCC staging system of NPC (2 for stage IIa, 24 for stage IIb, 16 for stage III, and 3 for stage IV) and displayed no other abnormalities on radiographic and serological examinations with a KPS of at least 70. All patients in this clinical study were able to complete the planned treatment without unexpected interruption.

After two cycles of inductive chemotherapy and a median duration of 40 days (range, 37–43 days) of radiation, tumor shrinkage presented in 24/45 (53.3%) of cases. Ten cases were defined as PR, with no cases of CR. After one month of adjuvant chemotherapy, 30/45 patients (66.7%) achieved CR, while 14/45 (31.1%) achieved PR, and 1/45 (2.2%) achieved SD. Although four patients with PR still had persistent regional disease, no hypermetabolic lesions were detectable by PET/CT. The response rate to the therapy regimen in our study was 97.8%.

All patients completed the follow-up regimen as planned, after 51 months of follow-up (range, 36–60 months). Follow-up data were collected and analyzed by a multidisciplinary team of radiologists and medical oncologists. The rates of OS and PFS were 95.5 and 93.3%, respectively, although three cases of treatment failure were documented. One of these cases, initially a stage III, developed multiple lung metastases at five months after adjuvant chemotherapy. The other two patients presented with progressive disease for multiple bone metastases and locoregional recurrence, respectively. These three patients subsequently received salvage radiotherapy, chemotherapy or surgery to attain an expectedly prolonged survival and higher quality of life.

### Treatment-related toxicities

The treatment-related toxic effects from the time of irradiation commencement to one month after irradiation are given in Table I. The toxicities, according to RTOG criteria, were listed. No patients had life-threatening or fatal toxicities related to chemotherapy or radiotherapy. Furthermore, no grade 4 toxicities were detected in our patient cohort. Although all the patients complained of dry mouth, this symptom was rapidly treated and relieved. At the completion of adjuvant chemotherapy, 8/45 cases (17.8%) had grade 1 toxicity, 27/45 (60.0%) had grade 2 toxicity, and 10/45 (22.2%) had grade 3 toxicity. Nevertheless, compared to conventional radio-chemotherapy regimens, the incidence of grade 3 toxicities was significantly decreased (P<0.05, 22.2 vs. 6.7%) and concomitant with an increase in grade 1 or 2 toxicity (P<0.05, 77.8 vs. 95.6%). Notably, conventional radio-chemotherapy-related side effects, including hepatotoxicity, nephrotoxicity, digestive disorders and weight loss, were severe at six months after irradiation (Table I). In this study, it was discovered that locoregional toxicities, such as pharyngitis, laryngitis, stomatitis, xerostomia and skin desquamation, were more frequent than other toxicities. Notably, six complete cycles of cisplatin-involving chemotherapy did not lead to serious hematological disorders (Table I).

## Discussion

The combination of chemotherapy with radiotherapy is a critical strategy for improving tumor control of locoregionally-advanced NPC, due to the potential enhancement of radiotherapy-mediated locoregional control and deracinating micro-metastasis. A meta-analysis of 1,753 patients from eight randomized trials has previously confirmed the added value of additional chemotherapy (16). Furthermore, the added benefit of cisplatin-based chemotherapy in combination with conventional-fractionation radiotherapy was demonstrated by the Intergroup 0099 Study (9). However, as the magnitude of long-term efficacy and safety is essential, the chemotherapeutic regimen of cisplatin plus 5-FU was subsequently recommended for advanced NPC.

A recent study on recurrent or metastatic head and neck cancer showed that, although the response rate to the combination of cisplatin and 5-FU was superior to single drugs, OS or PFS did not improve (21). It is possible, therefore, that the combination of cisplatin with 5-FU is not an effective combination for the eradication of micro-metastasis and affecting survival in head and neck cancers. Newer drugs, such as taxanes and gemcitabine, have exhibited promising results in NPC despite no improvement of OS (22,23). One strategy for improvement of NPC is, therefore, to add inductive or/and adjuvant chemotherapy before or/and after concurrent radio-chemotherapy. Neither of the two randomized studies of adjuvant chemotherapy given after radio-chemotherapy demonstrated an OS advantage over radiotherapy alone, however (6,15,21,24). Both of these studies had a major limitation, though, which was a significant rate of patient refusal to complete the planned adjuvant chemotherapy. Theoretically, the poor compliance with adjuvant chemotherapy after concurrent radio-chemotherapy can be overcome by the addition of inductive chemotherapy. A recent meta-analysis revealed a significant improvement of OS and PFS with cisplatin and 5-FU induction chemotherapy in patients with squamous cell carcinoma of the head and neck (20,25). Thus, new chemotherapy sequences that might improve the efficacy of chemotherapy as an adjunct should be further investigated.

In this study, all patients were assigned to receive two cycles of inductive chemotherapy (cisplatin plus 5-FU), two cycles of concurrent chemotherapy (cisplatin alone), and two cycles of adjuvant chemotherapy (cisplatin plus 5-FU). The patient compliance with all six cycles of chemotherapy and the full-course SMART-IMRT were of great significance. All patients recruited into this study accomplished the treatment as planned and no delays or dose reductions were demonstrated. This indicated that this treatment strategy had an acceptable compliance, so further improvements in tumor control were taken into account.

This regimen resulted in a notable response rate (97.8%) and nadir PD (CR of 66.7%, PR of 31.1%, and SD of 2.2%), which were similar to the response rate of early-stage NPC to radio-chemotherapy (7,26). Furthermore, OS and PFS in our study were significantly superior to those in previous reports on locoregionally-advanced NPC (5,27,28). Collectively, it implied that this regimen had potent tumor control of locoregionally-advanced NPC and an acceptable level of compliance. Our favorable results indicated that the use of the SMART-IMRT technique might lower the risk of toxicity of the organ and increase the curative effect.

Currently, IMRT is commonly chosen to treat NPC. IMRT is preferable to conventional 3D conformal planning as it further improves tumor coverage and dose distribution between the tumor and dose-limiting organs (27–29). Furthermore, IMRT also resolves the problem of dose uncertainty at the junction between the primary tumor and lymphatic regions, as it enables the primary tumor and the neck lymph nodes to be treated in one volume throughout (17,28). Recent studies supporting the superiority of IMRT over 2D-RT have reported a related 97% PFS and 88% OS at four years after IMRT, with a total of 65–70 Gy delivered to the GTV (30). Kam *et al* also reported that 3-year PFS and OS were 92 and 90%, respectively, with a total dose of 66 Gy. These studies testify to the efficacy of IMRT.

In addition to conformal dose distribution, IMRT can also be applied to exploit the therapeutic advantages of accelerated forms of radiotherapy. The acceleration scheme (involving multiple daily fractions, concomitant boosts and weekly six-daily treatments) improves tumor control and survival with increased but acceptable toxicities, irrespective of the acceleration schemes applied (7,28). The underpinning mechanism for the improvement of outcome is primarily due to a shortened overall treatment time and a reduction in the rate of tumor cell repopulation.

SMART acceleration techniques deliver different doses to different target volumes, simultaneously, through a fraction (29). Lauve *et al* reported the results of a phase I radiation dose-escalation trial to determine the maximal tolerable dose (MTD) of an accelerated fractionation with a simultaneous integrated boost for the treatment of locally advanced head and neck carcinoma (31). A total dose of 70.8 Gy by 30 fractions of 2.36 Gy was determined as the MTD deliverable to the GTV with adequate parotid sparing. The actuarial two-year locoregional control and distant control rates were 76.3% and 71.8%, respectively. It was concluded that tumor control and survival rates compared favorably with the outcomes of other accelerated regimens.

Despite reducing the amount of radiation received by non-target normal tissue, the application of accelerated RT by SMART also delivers a higher biologically effective dose to the normal mucosa within the target volume, which results in a higher prevalence of locoregional radiation-related diseases (such as orolarygopharyngeal mucositis and xerostoma) compared to conventionally fractionated RT (18,19,27,32–34). In this trial, all 45 patients recruited into the study were treated with SMART-IMRT and, as reported previously (18,35), orolaryngopharyngeal mucositis was more frequently observed than other toxic effects. While reported by almost all of the patients, grade 3 toxicity was only observed in 2/45 (4%) of patients and was quickly treated in all cases. With the parotid glands spared, grade 3 xerostomia was detected in 10/45 (22.2%) patients. Lee *et al* documented no chronic xerostomia (17), whereas Kam *et al* reported 23% of grade 2 or 3 xerostomia (7). However, the correlation between the salivary flow and subjective symptoms of xerostomia was relatively weak (7).

The interaction between SMART-IMRT and chemotherapy was analyzed. The addition of SMART-IMRT to chemotherapy in this study might have narrowed the potential gain in local control by the chemotherapy. Numerous phase III trials found that severe (grade 3) mucositis was more frequently associated with CRT than RT alone, with 37 to 62% vs. 28 to 48%, respectively (P<0.05) (35–37). Nevertheless, the toxic effects of concurrent radio-chemotherapy was well-tolerated, supported by the observation that all patients in our cohort accomplished six full cycles of inductive, concurrent and adjuvant chemotherapy as planned. Parallel with these outcomes, hematological and non-hematological toxic effects were ascribed to an acceptable and short-term modality and were not life-threatening. The outcomes of our trial therefore supported a regimen of six cycles of cisplatin-based chemotherapy concurrent with SMART-IMRT as an acceptable and feasible strategy for locoregionally-advanced NPC. It is possible that administering combinations of newer drugs before, rather than after, concurrent radio-chemotherapy to improve compliance might result in further improvements in systemic control. These encouraging results are currently being confirmed in several randomized trials assessing new combinations of inductive chemotherapy with subsequent concurrent radio-chemotherapy (14,23).

There are several potential bias factors of this study, including a small sample size and the relatively short follow-up period. Effort was made to make up for these limitations. The IMRT guidelines using SMART were implemented and SMART protocols were consistent over the entire study period. In addition, the clinical follow-up data were collected from all patients in the cohort. In conclusion, despite a small patient sample size and a short follow-up, the preliminary results demonstrate an encouraging trend of locoregional control and survival with no increase in related toxicities. Although we reported on two-year survival results, we are still following the patients closely and reporting five-year follow-up results when more events become available. The findings suggest that increasing the availability of SMART-IMRT may further increase the therapeutic gain. Estimation of sample size for future trials should be based on higher baseline results by SMART-IMRT and more realistic magnitude of benefit to avoid being underpowered.

## Figures and Tables

**Table I:** Toxicities according to treatment.

	Grade of toxic effects			
	I	II	III	IV
Skin	12	28	5	0
Mucosa	6	35	4	0
Salivary glands	8	27	10	0
Pharynx	35	10	0	0
Larynx	45	0	0	0
Digestive disorders	14	28	3	0
Vomiting	8	15	2	0
Nausea	14	20	2	0
Diarrhea	1	0	0	0
Hematological	23	9	6	0
Anemia	15	5	0	0
Leucocytopenia	23	9	6	0
Thrombocytopenia	1	0	0	0
Hepatotoxicity	2	0	0	0
Nephrotoxicity	0	0	0	0
Weight loss	10	30	5	0
